# Defining the learning curve of point-of-care ultrasound for confirming endotracheal tube placement by emergency physicians

**DOI:** 10.1186/s13089-015-0031-7

**Published:** 2015-09-17

**Authors:** Jordan Chenkin, Colin J. L. McCartney, Tomislav Jelic, Michael Romano, Claire Heslop, Glen Bandiera

**Affiliations:** Department of Emergency Medicine, Sunnybrook Health Sciences Centre, 2075 Bayview Avenue, Toronto, ON M4N 3M5 Canada; Division of Emergency Medicine, University of Toronto, 2075 Bayview Avenue C753, Toronto, ON M4N 3M5 Canada; Department of Anesthesiology, University of Ottawa, Room B311, 1053 Carling Avenue, Mail Stop 249, Ottawa, ON K1Y 4E9 Canada; Department of Emergency Medicine, St. Michael’s Hospital, 30 Bond Street, Toronto, ON M5B 1W8 Canada

**Keywords:** Endotracheal intubation, Endotracheal intubation confirmation, Ultrasonography, Education, Upper airway ultrasound

## Abstract

**Background:**

Unrecognized esophageal intubations are associated with significant patient morbidity and mortality. No single confirmatory device has been shown to be 100 % accurate at ruling out esophageal intubations in the emergency department. Recent studies have demonstrated that point-of-care ultrasound (POCUS) may be a useful adjunct for confirming endotracheal tube placement; however, the amount of practice required to become proficient at this technique is unclear. The purpose of this study is to determine the amount of practice required by emergency physicians to become proficient at interpreting ultrasound video clips of esophageal and endotracheal intubations.

**Methods:**

Emergency physicians and emergency medicine residents completed a baseline interpretation test followed by a 10 min online tutorial. They then interpreted POCUS clips of esophageal and endotracheal intubations in a randomly selected order. If an incorrect response was provided, the participant completed another practice session with feedback. This process continued until they correctly interpreted ten consecutive ultrasound clips. Descriptive statistics were used to summarize the data.

**Results:**

Of the 87 eligible physicians, 66 (75.9 %) completed the study. The mean score on the baseline test was 42.9 % (SD 32.7 %). After the tutorial, 90.9 % (60/66) of the participants achieved proficiency after one practice attempt and 100 % achieved proficiency after two practice attempts. Six intubation ultrasound clips were misinterpreted, for a total error rate of 0.9 % (6/684). Overall, the participants had a sensitivity of 98.3 % (95 % CI 96.3–99.4 %) and specificity of 100 % (95 % CI 98.9–100 %) for detecting correct tube location. Scans were interpreted within an average of 4 s (SD 2.9 s) of the intubation.

**Conclusions:**

After a brief online tutorial and only two practice attempts, emergency physicians were able to quickly and accurately interpret ultrasound intubation clips of esophageal and endotracheal intubations.

**Electronic supplementary material:**

The online version of this article (doi:10.1186/s13089-015-0031-7) contains supplementary material, which is available to authorized users.

## Background

Emergency intubations are associated with a significant risk of esophageal intubation, which can be rapidly fatal if not recognized and corrected quickly [1]. Qualitative color capnography is commonly used to help to confirm endotracheal tube position; however, it has been shown to be indeterminate or unreliable in a significant proportion of emergency department patients [2]. In addition, capnography requires that ventilations be delivered to the patient which can increase the risk of aspiration if the tube is misplaced in the esophagus. No single airway confirmation device has been shown to be 100 % accurate in all patient scenarios; therefore, emergency physicians should consider the use of multiple confirmation techniques to reduce the risk of an unrecognized esophageal intubation.

Recently, there has been increasing evidence supporting the use of point-of-care ultrasound (POCUS) for confirmation of endotracheal tube placement. Ultrasound can be used to identify the endotracheal tube position in real time with a high degree of accuracy and without the need for any ventilations being delivered to the patient [3–14]. Two recent meta-analyses found a combined sensitivity of 93–98 % and specificity of 97–98 % for the use of ultrasound for confirming endotracheal tube location [15, 16]. A strategy of combining POCUS and capnography may significantly reduce the risk of an esophageal intubation going undetected and prevent the associated patient morbidity and mortality [9].

Despite the increasing evidence to support the use of POCUS for confirming endotracheal intubation, there is limited data to guide training protocols for this technique. Specifically, the required amount and optimal format of training as well as the number of scan interpretations required to achieve competence with the technique have not been evaluated. Since the consequences of misinterpreting an intubation ultrasound could be devastating, it is important that any training protocol is sufficient for the learners to achieve a very high accuracy at image interpretation. The objective of this study is to determine the amount of practice required by emergency physicians to achieve proficiency with interpretation of POCUS video clips of endotracheal tube placement.

## Methods

### Study design

We conducted a prospective educational study to evaluate the learning curve of interpreting POCUS video clips for endotracheal intubation. Using the principles of effective Web-based educational material development, we developed an online educational module covering the technique of using ultrasound for confirming endotracheal intubation [17]. We designed an online assessment tool that allowed us to track the number of practice attempts and correct and incorrect interpretations from each participant. The educational module and assessment tool were pilot tested by several physicians with experience in the technique and modifications were made based on their feedback. The study was approved by the institutional Research Ethics Board and each participant provided informed consent.

### Study setting and population

The study was performed between July and October 2014. All emergency physicians from a single academic emergency department and emergency medicine residents from the associated university program were invited to participate, regardless of their prior experience level with POCUS. The emergency department has an active ultrasound program; however, there is significant variation in its clinical use among the emergency physicians. The residency training program includes a 1-month rotation in emergency ultrasound; however, the curriculum does not include any training on the use of ultrasound for endotracheal tube confirmation.

**Fig. 1:**
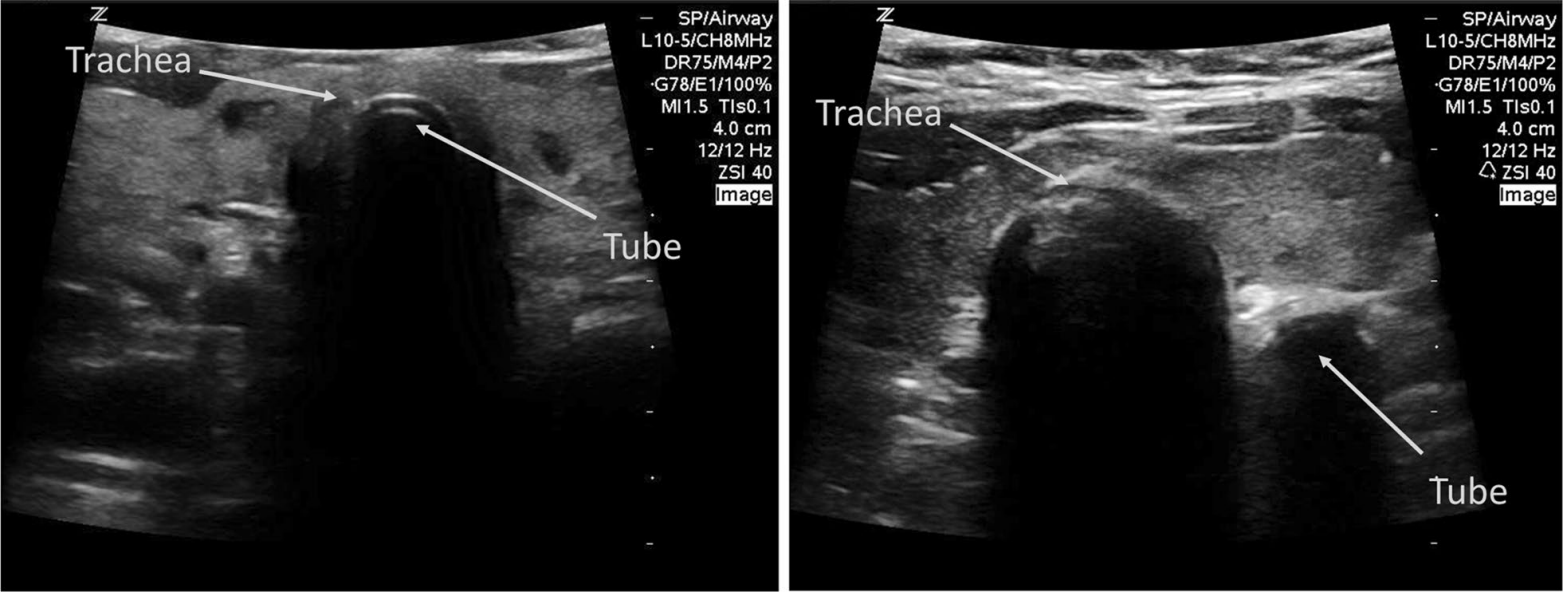
Ultrasound images of endotracheal and esophageal intubations. Images are generated by placing a high-frequency linear transducer in the transverse position (indicator facing the patient’s *right side*) at the level of the suprasternal notch. On the *left image*, a normal endotracheal intubation is shown with the echogenic semicircular tube visible within the lumen of the trachea. On the *right image*, an esophageal intubation can be identified by the presence of a ‘double tract sign’ with a second semi-circular acoustic shadow appearing outside of the trachea

To develop the test bank of ultrasound video clips, we enrolled a convenience sample of patients undergoing elective surgery requiring endotracheal intubation. We selected patients with a broad spectrum of demographics, body habitus, and difficulty of intubation (Table 1). After anesthesia induction, an endotracheal tube was passed first into the esophagus and then into the trachea under direct visualization. A total of 20 esophageal and 20 tracheal intubation clips were captured by a study investigator (JC) using a Zonare Z. One ultrasound machine (ZONARE Medical Systems, Inc, Mountain View CA) with a 10–5 MHz linear array transducer in the transverse position at the suprasternal notch. Clip recording started upon passage of the endotracheal tube into the mouth and stopped once inserted fully to the appropriate depth. To increase the generalizability of our study results, we included all saved clips regardless of scan difficulty or quality. Typical images of tracheal and esophageal intubations are shown in Fig. 1 (see Additional files 1 and 2 for example video clips). All endotracheal tube positioning was confirmed using quantitative waveform capnography.

**Table 1:** Demographics of intubated patients

	*N* = 20
Age, mean (year)	64.1 ± 15.2
Female sex, no. (%)	11 (55)
Weight, mean (kg)	80.1 ± 13.3
Height, mean (cm)	171 ± 10.4
BMI, mean (kg/m^2^)	27.3 ± 3.3
Difficult airway, no. (%)	4 (20)

### Study protocol

Emergency physicians and residents were invited to participate in the study by email invitations sent by a research coordinator. Participants were directed to an online tutorial where they first completed a baseline questionnaire and a baseline image interpretation test consisting of five esophageal and five tracheal intubation videos in random sequence. For each video clip, participants were asked whether the tube was placed in the trachea or the esophagus. Following this baseline test, participants completed a 10-min Web-based tutorial which covered the background of POCUS for confirming endotracheal intubation, ultrasound technique, image interpretation, and common pitfalls. Our tutorial included commonly accepted ultrasound findings for tracheal intubation (fluttering movement of the tube within the trachea and no change in the appearance of the esophagus) and esophageal intubation (appearance of a ‘double tract’ second shadow adjacent to the trachea). Following the tutorial, the participants completed a single practice interpretation with specific feedback provided based on their response.

To evaluate the learning curve, participants then completed an online test asking for interpretation of ultrasound clips of esophageal and endotracheal intubations selected in a random order. If the clip was correctly interpreted, they proceeded to the next clip. If the clip was incorrectly interpreted, the participant was required to complete more practice with feedback. No clip was seen more than one time per participant. This process continued until the participant achieved ten correct interpretations in a row. We selected ten sequential correct interpretations as our end point based on pilot testing with several staff, which suggested that the number of interpretations required to achieve proficiency was significantly less than ten. Following the assessment, a questionnaire was completed to assess participants’ comfort level with the technique.

### Measures

Self-reported comfort scores were measured using an anchored 5-point Likert scale. The assessment protocol was scored based on the number of practice sessions required, as well as the amount of time required to interpret the scans. Clip interpretation time was measured from when the intubation clip first started playing until interpretation was completed.

### Data analysis

Data were analyzed using Microsoft Excel (Microsoft Corp, WA, USA). Descriptive statistics were used to report pre-test scores, number of practice attempts, and time to interpretation. Sensitivity and specificity of image interpretation were calculated along with their 95 % confidence intervals by pooling all of the interpretations performed in the assessment phase of the study. Comfort scores were compared using the Wilcoxon signed-rank test using a two-tailed *p* value.

## Results

A total of 87 subjects were invited to participate in the study, including 36 emergency physicians and 51 emergency medicine residents. Of those, 25 (69.4 %) emergency physicians and 41 (80.4 %) emergency medicine residents completed the study, for an overall participation rate of 66/87 (75.9 %). Characteristics and prior ultrasound experience of the study participants are listed in Table 2. The majority of participants (81.8 %) had no previous experience with using ultrasound for confirming endotracheal tube position. The mean score on the baseline intubation ultrasound test was 42.9 % (SD 32.7 %).

**Table 2:** Demographics of study participants

	N = 66 (%)
Staff physician	25 (37.9)
Resident physician	41 (62.1)
Resident—PGY-1	9 (13.6)
Resident—PGY-2	7 (10.6)
Resident—PGY-3	9 (13.6)
Resident—PGY-4	7 (10.6)
Resident—PGY-5	9 (13.6)
Basic ultrasound certification	45 (68.2)
Attended advanced ultrasound course	18 (27.3)
Previous intubation ultrasound experience	12 (18.2)

*PGY* postgraduate year

**Fig. 2:**
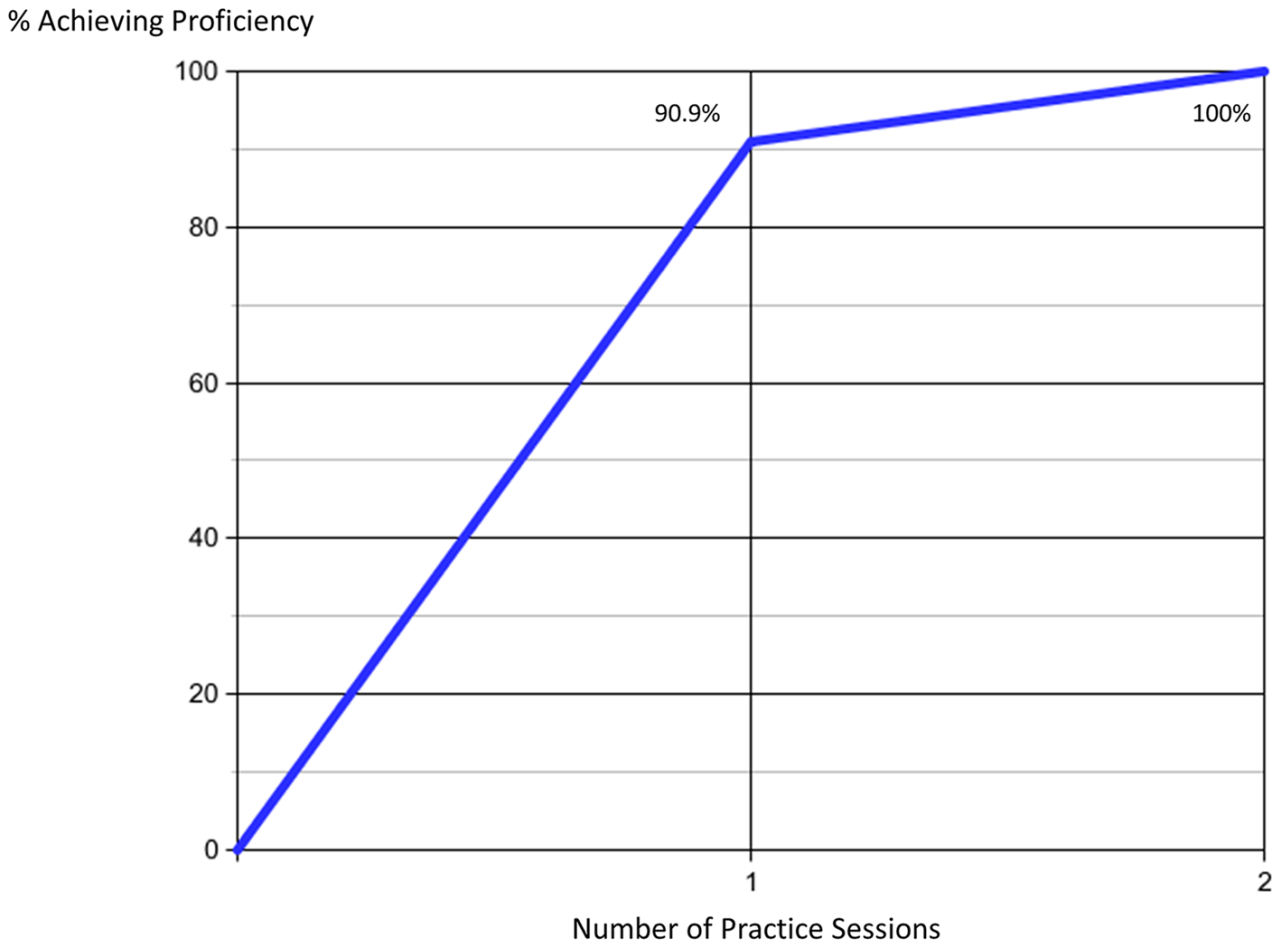
Learning curve for intubation ultrasound. The cumulative percentage of participants achieving proficiency is shown based on the number of practice sessions completed

The number of practice attempts required to achieve proficiency after completing the tutorial is displayed in Fig. 2. Overall, 90.9 % (60/66) achieved proficiency after only a single practice attempt. Six participants (9.1 %) made an incorrect interpretation after the first practice and required a second practice session with additional feedback. All six participants were successful after the second practice session, and no participants required more than two practice attempts. All six errors were due to tracheal intubations being misidentified as esophageal placements. The number of correct interpretations before making a mistake ranged between three to six clips.

After completing the 10-min online tutorial, the participants had an overall sensitivity of 98.3 % (95 % CI 96.3–99.4 %) and specificity of 100 % (95 % CI 98.9–100 %) for detecting correct tube location using ultrasound video clips. The overall accuracy for ultrasound interpretation was 99.1 % (678/684). The mean amount of time from the beginning of the intubation clip to the interpretation was 11 s (SD 2.8 s). After subtracting the time for the intubation itself, the interpretation was completed within an average of 4 s (SD 2.8 s) of the intubation. Participants reported a significant improvement in their comfort in image generation and interpretation before and after the tutorial (Table 3).

**Table 3:** Comfort levels with image interpretation and performance of ultrasound scan before and after the tutorial

	Baseline	Post-tutorial	*p* value
Comfortable or very comfortable with interpretation of images	1/66 (1.5 %)	63/66 (95.5 %)	<0.001
Comfortable or very comfortable with performance of technique	1/66 (1.5 %)	48/66 (72.7 %)	<0.001

## Discussion

The use of point-of-care ultrasound for confirming endotracheal tube placement is a relatively new, yet promising technique that may help to avoid the morbidity associated with unrecognized esophageal intubations. With any new procedure where the learning curve is not yet defined, it is important to understand the amount of practice required to achieve competence. There is a wide variation in learning curves for the various point-of-care ultrasound techniques used by emergency physicians. Some scans require a significant amount of practice, whereas others are quick to master [18]. With endotracheal tube confirmation, any new technique must be easy to learn, and results must be able to be interpreted quickly with a high degree of accuracy.

The results from this study demonstrate that after a brief online tutorial and only one or two practice sessions with feedback, emergency physicians can quickly and accurately interpret ultrasound videos of esophageal and tracheal intubations. There are several possible reasons that may be contributing to the relatively short learning curve for this technique. Unlike many other emergency ultrasound applications, the transducer is placed in a uniform location and once placed does not need to move. The anatomy is relatively consistent between patients and is easy to identify. Body habitus plays less of a factor than in abdominal scans, as even in obese patients the airway anatomy is easily visible on ultrasound.

Previous studies have demonstrated that point-of-care ultrasound is very accurate at differentiating esophageal from tracheal tube placement. However, many of these studies are limited to including very few sonologists or by having the investigators themselves performing the ultrasound scans [3–5, 8, 9, 12, 19]. This limits the generalizability of the results and provides no information on whether the scan technique can be performed by emergency physicians with limited experience with ultrasound for intubation.

Other intubation ultrasound studies that included non-expert sonologists reported accuracy levels similar to our study findings after only brief training interventions. Goksu et al. found that after a 15-min presentation, seven physicians achieved an overall sensitivity of 95.7 % and specificity of 98.2 % [20]. Ma found that after a 5-min briefing, seven residents achieved a sensitivity of 97 % and specificity of 100 % on cadaver models [21]. Uya et al. found that after a 20-min didactic session and a 30-min practice session, eight novice pediatric emergency medicine fellows achieved a sensitivity of 96 % for tracheal location [22]. These studies were limited by the small numbers of participants and the use of a cadaver model for the intubations.

One previously published study attempted to evaluate the level of difficulty of endotracheal tube localization using ultrasound [23]. In this study, 29 participants with different levels of general point-of-care ultrasound experience identified endotracheal tube location on cadavers. In this study, experienced sonologists (defined as having >150 scans) demonstrated a higher sensitivity and specificity compared with less experienced sonologists (sensitivity 75.0 vs 62.0 %, specificity 62.5 vs 37.9 %). This study was limited by the use of cadaver models and a static technique after the intubation was completed, which may have led to the relatively poor accuracy [21].

Our study adds to the previously published literature in several ways. First, we included a large cohort of staff and resident physicians with a broad range of ultrasound experience from complete novices to some who have completed an ultrasound fellowship. Unlike many of the previous studies, we used live patient intubations as opposed to cadaver models. We also included patients with a wide range of body habitus and scan difficulty ranging from easy to difficult to interpret. Our study is the first to use repeated practice with tailored feedback to determine the learning curve for interpreting intubation ultrasound clips. Despite the variation in experience level and patient population, we found that all participants were highly accurate with their ultrasound interpretations after only two practice attempts with feedback.

All of the clip misinterpretations in our study were due to tracheal intubations thought to be in the esophagus. While not as dangerous as missing an esophageal intubation, this error may still result in patient harm if a correctly placed tube is unnecessarily removed. The findings of this study fit with our real-world experience, where the fluttering movement seen with correct tube placement is more subtle than the double tract sign of an esophageal intubation. To avoid this pitfall, one strategy is to locate the esophagus before the intubation and observe if it changes during the passage of the tube. If it does not, the tube is located in the trachea. If a second shadow appears where the esophagus lies, the tube is in the esophagus. Another important pitfall which was not seen in this study was the esophagus that lies posterior to the trachea. In this situation, identification of an esophageal tube can be difficult. This pitfall can be avoided by keeping the transducer placement as low as possible on the neck and applying slight pressure to help bring the esophagus into view.

This study has several limitations. For logistical reasons, we only evaluated image interpretation and not image generation. In actual practice, emergency physicians must be able to simultaneously generate and interpret images to make clinical decisions in real time. However, we have found that generating ultrasound images for endotracheal tube confirmation is relatively easy compared with other ultrasound techniques. Our test bank of ultrasound clips was relatively small, consisting of 20 esophageal and 20 tracheal intubations. However, we included clips taken from patients with a broad range of neck anatomy and difficulty of intubation. We selected ten consecutive correct interpretations as a marker for proficiency, and it is possible that a larger sample would have revealed different results. However, we noted that the vast majority of participants were successful after only one practice attempt, supporting our theory that these images are relatively easy to learn. It seems unlikely that many more cases would have made a difference in the study findings. For logistical reasons, the tutorial and evaluations were completed on the same day. Ideally, a washout period would be preferred to test knowledge retention. Finally, the ultrasound clips taken of elective intubations in the controlled environment of the operating room may not be generalizable to emergency department intubations, where images may be more difficult to generate or interpret.

## Conclusions

In this study, we found that emergency physicians were able to accurately interpret ultrasound clips for endotracheal intubation after a brief online tutorial and only two practice attempts. Given that image interpretation appears to be easily learned, this technique may be a useful adjunct to other airway confirmation devices in the emergency department. Future studies are needed to determine the learning curve for airway image generation and to confirm these findings in emergency department intubations.

## Additional files

Additional file 1: Video demonstrating the appearance of a normal endotracheal intubation using ultrasound. Additional file 2: Video demonstrating the appearance of an esophageal intubation using ultrasound.

## References

[1] Cook TM, Woodall N, Harper J, Benger J, Fourth National Audit P (2011). Major complications of airway management in the UK: results of the Fourth National Audit Project of the Royal College of Anaesthetists and the Difficult Airway Society. Part 2: intensive care and emergency departments. Br J Anaesth.

[2] Li J (2001). Capnography alone is imperfect for endotracheal tube placement confirmation during emergency intubation. J Emerg Med.

[3] Chou HC, Chong KM, Sim SS, Ma MH, Liu SH, Chen NC, Wu MC, Fu CM, Wang CH, Lee CC, Lien WC, Chen SC (2013). Real-time tracheal ultrasonography for confirmation of endotracheal tube placement during cardiopulmonary resuscitation. Resuscitation.

[4] Adi O, Chuan TW, Rishya M (2013). A feasibility study on bedside upper airway ultrasonography compared to waveform capnography for verifying endotracheal tube location after intubation. Crit Ultrasound J.

[5] Chou HC, Tseng WP, Wang CH, Ma MH, Wang HP, Huang PC, Sim SS, Liao YC, Liao YC, Chen SY, Hsu CY, Yen ZS, Chang WT, Huang CH, Lien WC, Chen SC (2011). Tracheal rapid ultrasound exam (T.R.U.E.) for confirming endotracheal tube placement during emergency intubation. Resuscitation.

[6] Galicinao J, Bush AJ, Godambe SA (2007). Use of bedside ultrasonography for endotracheal tube placement in pediatric patients: a feasibility study. Pediatrics.

[7] Marciniak B, Fayoux P, Hebrard A, Krivosic-Horber R, Engelhardt T, Bissonnette B (2009). Airway management in children: ultrasonography assessment of tracheal intubation in real time?. Anesth Analg.

[8] Milling TJ, Jones M, Khan T, Tad-y D, Melniker LA, Bove J, Yarmush J, SchianodiCola J (2007). Transtracheal 2-d ultrasound for identification of esophageal intubation. J Emerg Med.

[9] Park SC, Ryu JH, Yeom SR, Jeong JW, Cho SJ (2009). Confirmation of endotracheal intubation by combined ultrasonographic methods in the Emergency Department. Emerg Med Aust EMA.

[10] Pfeiffer P, Rudolph SS, Borglum J, Isbye DL (2011). Temporal comparison of ultrasound vs. auscultation and capnography in verification of endotracheal tube placement. Acta Anaesthesiol Scand.

[11] Weaver B, Lyon M, Blaivas M (2006). Confirmation of endotracheal tube placement after intubation using the ultrasound sliding lung sign. Acad Emerg Med Off J Soc Acad Emerg Med.

[12] Werner SL, Smith CE, Goldstein JR, Jones RA, Cydulka RK (2007). Pilot study to evaluate the accuracy of ultrasonography in confirming endotracheal tube placement. Ann Emerg Med.

[13] Abbasi S, Farsi D, Zare MA, Hajimohammadi M, Rezai M, Hafezimoghadam P (2015). Direct ultrasound methods: a confirmatory technique for proper endotracheal intubation in the emergency department. Eur J Emerg Med Off J Eur Soc Emerg Med.

[14] Hoffmann B, Gullett JP, Hill HF, Fuller D, Westergaard MC, Hosek WT, Smith JA (2014). Bedside ultrasound of the neck confirms endotracheal tube position in emergency intubations. Ultraschall Med.

[15] Das SK, Choupoo NS, Haldar R, Lahkar A (2014). Transtracheal ultrasound for verification of endotracheal tube placement: a systematic review and meta-analysis. Can J Anaesth.

[16] Chou EH, Dickman E, Tsou PY, Tessaro M, Tsai YM, Ma MH, Lee CC, Marshall J (2015). Ultrasonography for confirmation of endotracheal tube placement: a systematic review and meta-analysis. Resuscitation.

[17] Cook DA, Dupras DM (2004). A practical guide to developing effective web-based learning. J Gen Intern Med.

[18] Blehar DJ, Barton B, Gaspari RJ (2015). Learning curves in emergency ultrasound education. Acad Emerg Med Off J Soc Acad Emerg Med.

[19] Muslu B, Sert H, Kaya A, Demircioglu RI, Gozdemir M, Usta B, Boynukalin KS (2011). Use of sonography for rapid identification of esophageal and tracheal intubations in adult patients. J Ultrasound Med Off J Am Inst Ultrasound Med.

[20] Goksu E, Sayrac V, Oktay C, Kartal M, Akcimen M (2010). How stylet use can effect confirmation of endotracheal tube position using ultrasound. Am J Emerg Med.

[21] Ma G, Davis DP, Schmitt J, Vilke GM, Chan TC, Hayden SR (2007). The sensitivity and specificity of transcricothyroid ultrasonography to confirm endotracheal tube placement in a cadaver model. J Emerg Med.

[22] Uya A, Spear D, Patel K, Okada P, Sheeran P, McCreight A (2012). Can novice sonographers accurately locate an endotracheal tube with a saline-filled cuff in a cadaver model? A pilot study. Acad Emerg Med Off J Soc Acad Emerg Med.

[23] Stuntz R, Kochert E, Kehrl T, Schrading W (2014). The effect of sonologist experience on the ability to determine endotracheal tube location using transtracheal ultrasound. Am J Emerg Med.

